# Medication use and driving patterns in older drivers: preliminary findings from the LongROAD study

**DOI:** 10.1186/s40621-020-00265-y

**Published:** 2020-08-03

**Authors:** Linda L. Hill, Howard Andrews, Guohua Li, Carolyn G. DiGuiseppi, Marian E. Betz, David Strogatz, Patricia Pepa, David W. Eby, David Merle, Tara Kelley-Baker, Vanya Jones, Samantha Pitts

**Affiliations:** 1 Department of Family Medicine and Public Health, University of California, 200 W Arbor Dr., MC 0811, San Diego, CA 92103 USA; 2 Department of Biostatistics, Mailman School of Public Health, Columbia University, 1051 Riverside Dr. Unit 47, New York, NY 10032 USA; 3 Department of Epidemiology, Mailman School of Public Health, Columbia University, 722 W 168th St. Rm 524, New York, NY 10032 USA; 4 Center for Injury Epidemiology and Prevention, Columbia University Medical Center, 722 W 168th St. Rm 524, New York, NY 10032 USA; 5 Department of Anesthesiology, Vagelos College of Physicians and Surgeons, Columbia University, 722 W 168th St. Rm 524, New York, NY 10032 USA; 6 Department of Epidemiology, Colorado School of Public Health, 13001 E. 17th Place, Mail Stop B119, Bldg. 500, Rm. W3138, Aurora, CO 80045 USA; 7 Department of Emergency Medicine, University of Colorado School of Medicine, Anschutz Medical Campus, Leprino Building, Campus Box B215, 12401 East 17th Ave, Aurora, CO 80045 USA; 8 Bassett Research Institute, Bassett Healthcare Network, 1 Atwell Rd, Cooperstown, NY 13326 USA; 9 Department of Ambulatory Care Clinical Pharmacy, Kaiser Permanente, Oakland, USA; 10 Transportation Research Institute, University of Michigan, 2901 Baxter Rd, Ann Arbor, MI 48109 USA; 11 AAA Foundation for Traffic Safety, 607 14th St. NW, Ste. 201, Washington, DC 20005 USA; 12 Johns Hopkins Bloomberg School of Public Health, 624 North Broadway, Hampton House, Baltimore, MD 21205 USA; 13 Department of Medicine, School of Medicine, Johns Hopkins University, 733 North Broadway, Baltimore, MD 21205 USA

**Keywords:** Older adult, Driving safety, Older driver, Medication, Driving outcome

## Abstract

**Background:**

The potential for impaired driving due to medication use can occur at any age, though older adults are more likely to take multiple prescribed medications and experience side effects that may affect driving ability. The purpose of this study was to characterize the relationship between medications and driving safety behaviors.

**Methods:**

Data for this study came from the five-site Longitudinal Research on Aging Drivers (LongROAD) project. Participants were active drivers, age 65–79 years at enrollment, and patients at one of the 5 participating sites. Medication names and doses were obtained at baseline based on the “brown-bag review” method. Medications were coded using the American Hospital Formulary Service system. Driving data were collected by a GPS accelerometer installed in the study participants’ main vehicles.

**Results:**

Medication data were available for 2949 (98.6%) of the 2990 participants, and 2898 (96.9% of all participants) had both medication data and at least 30 recorded days of driving. The median number of medications taken per study participant was seven, with a range of 0–51. Total number of medications was significantly associated with a higher rapid deceleration rate. Certain medication classes were significantly associated with other driving outcomes, including central nervous system agents (more speeding events), hormones and gastrointestinal medications (more rapid decelerations), electrolytes (fewer rapid decelerations), and antihistamines (greater right to left turn ratio).

**Conclusions:**

Older adult drivers are taking large quantities of prescription and non-prescription medications that may affect their driving safety. Certain medication classes are associated with potentially adverse driving patterns, such as speeding and rapid decelerations, while others are associated with potentially protective maneuvers, such as right hand turning. Further research is warranted to identify and mitigate potential adverse effects of such medications on driving safety in older adults.

## Background

Motor vehicles crashes are a major cause of death in the US with 36,560 fatalities in 2018 (National Highway Traffic Safety Administration 2019). Prescribed medications, over the counter (OTC) medications, and recreationally used drugs, including alcohol, have the potential to interfere with the ability to drive safely (Ray et al. 1993; National Highway Traffic Safety Administration 2008; National Highway Traffic Safety Administration 2010; Hetland and Carr 2014), with the risk increasing with the number of medications (Monárrez-Espino et al. 2014). A number of experimental and epidemiological studies, as well as comprehensive reviews, have indicated potential increases in crash risk associated with several classes of drugs (Orriols et al. 2009; Orriols et al. 2010; Gjerde et al. 2015; Strand et al. 2016). The reported list of impairing drugs includes: analgesics, central nervous system (CNS) drugs, antihistamines, antidepressants, and others (McGwin et al. 2000; Dubois et al. 2008; Brunnauer et al. 2004; Weiler et al. 2000; Verster and Volkerts 2004; Hill et al. 2017; Dassanayake et al. 2011; Engeland et al. 2007; Dubois et al. 2010). In a meta-analysis of 27 studies, Rudisill et al. found that 15 (28.3%) of the 53 medications studied were associated with an increased risk of a motor vehicle crash (MVC) (Rudisill et al. 2016a). Notably, many of these medications were benzodiazepines and narcotic pain medications, classes that are more commonly studied than other medication classes in both driving assessment and simulation studies.

The potential for impaired driving due to medications can occur at any age, though older adults are more likely to be taking prescribed medication, including using multiple pharmaceuticals with potential drug interactions. Kelley-Baker et al. found that 20% of drivers had used at least one prescription drug in the last 2 days (Kelley-Baker et al. 2017), with increased use at older age, and multiple other studies have documented high medication usage rates in the older adult population (Gurwitz et al. 2003; Rosenbloom and Santos 2014; Kaufman et al. 2002). Similarly, a survey of drivers 55 years and older found that 69% currently used one or more potentially impairing prescription medications, while 10% currently used at least five (MacLennan et al. 2009). Finally, with changes in metabolism and drug clearance (Klotz 2009), older drivers may also be more likely to experience prolonged side effects, thereby affecting their driving ability for longer periods of time compared with younger drivers. Side effects that impair driving skills may include, but are not limited to: drowsiness, confusion, low blood pressure, low blood sugar, nausea, loss of consciousness, weak muscle tone, and poor coordination. Despite these concerns, the crash rate of older drivers is less than their younger counterparts, though they have higher fatality rates (Palumbo et al. 2019; Insurance Institute for Highway Safety HLDI Fatality Facts 2018).

Certain medication classes have been associated with crash risk in older drivers. For example, Chihuri and Li found that the presence of opioids in fatal crashes increased from 1.0% in 2007 to 7.2% in 2015 (Chihuri and Li 2017); of the three studies in this analysis that assessed drivers 50 years or older, two of them found a statistically significant association between opioid use and crashes in this older population. Other medications associated with a greater crash risk include psychoactive medications (Meuleners et al. 2011), zolpidem (a sedative-hypnotic) (Booth et al. 2016), benzodiazepines (Rudisill et al. 2016a) and tramadol (Rudisill et al. 2016b).

Finally, as older drivers may also drink alcohol, interactions between medication and alcohol is a concern. Among participants of the National Survey on Drug Use and Health (NSDUH) aged 65 years and over, 52.2% reported using alcohol or illicit drugs (including cannabis) in the last 12 months (Choi et al. 2016). In a study of 404 Driving Under the Influence (DUI) conviction cases with drivers over the age of 70 years, 60% regularly used medications (Kirsch et al. 2017). Another study of suspected impaired older drivers reported the prevalence of alcohol (81%), benzodiazepines (15%) and z-narcotics (13%) based on blood tests (Høiseth et al. 2017).

While the effect of polypharmacy and certain medication classes on driving performance is established, few studies provide comprehensive medication reviews combined with objective driving data. The purpose of this study was to establish baseline methods for coding medications, and describe, in a large cohort of drivers aged 65–79 years, the associations between medication use and multiple driving outcomes.

## Methods

This study uses data from the multi-site Longitudinal Research on Aging Drivers (LongROAD) cohort study, described in detail elsewhere (Li et al. 2017). The LongROAD study aims are to explore the role of medical, behavioral, social, technological, and environmental factors in safe driving among older adults. The study enrolled older drivers in sites in five states (Ann Arbor, MI; Baltimore, MD; Cooperstown, NY; Denver, CO; and San Diego, CA). LongROAD collects self-reported and objectively-measured health and driving data (from a data-logger device collecting global positioning, accelerometer measurements, etc.), medical record information, medication history, and state motor vehicle driving records.

For study recruitment, invitations were sent by mail and e-mail to patients identified through electronic medical records of health care systems and clinics affiliated with the LongROAD sites. Potential participants were contacted by telephone for additional eligibility screening and recruitment. Individuals were eligible if they: were aged 65–79 years; possessed a valid driver’s license; drove on average at least once per week; had no significant cognitive impairment; drove a primary car (utilized ≥80% of the time) that was a 1996 model or newer (for the purposes of the monitoring device); were not already enrolled in any program involving installation of a driving behavior monitoring device in their primary vehicle; lived in the study site area at least 10 months out of the year, and had no plans to move to another city within 5 years; and were not married to or living with a current LongROAD participant. Eligible and interested individuals scheduled an in-person session, during which full written informed consent was obtained. Enrollment occurred between July 2015 and March 2017. Each site’s respective Institutional Review Board gave study approval.

### Measures

For the in-person enrollment session, participants were instructed to bring in all their medications, prescription and non-prescription, for a ‘brown bag medication review.’ All the medications and doses in the ‘brown bag’ were entered into the online database. The medications were then coded based on the American Hospital Formulary Service (AHFS) system (American Hospital Formulary Service AHFS Pharmacologic-Therapeutic Classification 2019). The AHFS classification allows the grouping of drugs with similar pharmacologic, therapeutic, and/or chemical characteristics in a 4-tier hierarchy, with each tier of classification providing additional detail about the effect of any given medication. There are 31 possible classifications in the first tier, 189 in the second tier, 269 in the third tier, and 105 in the fourth tier.

The AHFS classifications have the following advantages: 1) providing a standardized way of classifying drug use in this study sample, for purposes of comparison with other populations; 2) establishing meaningful categories for use in analyses examining the effect of medication on driving behavior and cognitive factors, as measured by self-reports, standardized assessment instruments and GPS data generated by the cars the participants drive; 3) adjusting for the effects of medication in regression models, when assessing the effects of other behavioral and vehicle-related factors on driving outcomes, including crashes, violations and dangerous driving practices; 4) tracking the effects of changes in medication use over time, including changes in the number, dosage and variety of medications with aging; and 5) facilitating the search for specific medications of interest in future analyses.

Participants’ medications were classified by a clinical psychiatric pharmacist. Non-classified medications included food-like items (e.g. spices, protein), homeopathic products, and other supplements (e.g. witch-hazel, Ginkgo). If an individual medication included more than one drug, each drug was classified separately. Similarly, some drugs met criteria for two or more classifications (i.e., the same drug may have two different Tier 1 classifications). In these instances, each classification was treated as a different medication. Where apparent spelling errors occurred in the entering of drug names, the most likely drug name was coded; there were no cases of ambiguity about intended drug names. Only the first tier of classifications was considered during this analysis (for example, within the Tier I label “Cardiovascular drugs,” Labetalol can also be classified under: Class II anti-arrhythmics, alpha-adrenergic blocking agents, and beta-adrenergic blocking agents; for this analysis, Labetalol is considered a “Cardiovascular drug”). A list of the pertinent Tier I classifications can be found in Table 1.

**Table 1:** Frequency of Tier 1 medication use by total medication and participant

	Frequency of medications used (*N* = 24,697 medications)^a^		Number of participants using each medication (*N* = 2949 participants)^b^	
	N	%	N	%
Cardiovascular Drugs	4700	19.0	2167	73%
Vitamins	3922	15.9	1909	65%
Central Nervous System Agents	3921	15.9	2078	70%
Electrolytic, Caloric, and Water Balance	2355	9.5	1548	52%
Hormones and Synthetic Substitutes	2189	8.9	1311	44%
Gastrointestinal Drugs	1219	4.9	952	32%
Eye, Ear, Nose, and Throat (EENT) Preparations	1047	4.2	710	24%
Autonomic Drugs	834	3.4	641	22%
Skin and Mucous Membrane Agents	658	2.7	486	16%
Blood Formation, Coagulation, and Thrombosis Agents	450	1.8	390	13%
Antihistamine Drugs	436	1.8	392	13%
Miscellaneous Therapeutic Agents	419	1.7	379	13%
Anti-infective Agents	353	1.4	291	10%
Respiratory Tract Agents	119	.5	106	4%
Antineoplastic Agents	116	.5	112	4%
Smooth Muscle Relaxants	90	.4	89	3%
Local Anesthetics	27	.1	25	1%
Diagnostic Agents	5	.0	5	< 1%
Antitoxins, Immune Globulins, Toxoids, and Vaccines	3	.0	3	< 1%
Not Classified	1834	7.4	989	34%

^a^ Note that many participants reported taking more than one drug belonging to the same Tier 1 category; as a result, total medications reported for each category may exceed the number of LongROAD participants

^b^ Note that a given participant may take medications in 2 or more categories

Multiple driving outcomes were obtained from a GPS accelerometer inserted into the OBD-port of participants’ main automobile, as described elsewhere (Li et al. 2017). This system automatically recorded all driving when the vehicle was turned on and could determine if the driver was the participant. The outcome measures for this report include rapid decelerations (number of deceleration events ≥0.35 g per 1000 miles driven), a measure of hard braking, speeding (number of speeding events > 80mph for at least 8 s per 1000 miles driven), and right-to-left turn ratios, a measure that can indicate avoidance of potentially risky situations (i.e., left turns across oncoming traffic, with a higher number indicating avoidance of left turns) (National Highway Traffic Safety Administration 2009; Karthaus and Falkenstein 2016; Chevalier et al. 2017; Chevalier et al. 2016). Additional GPS measures were total miles driven and total days the participant drove. The medication, demographic and covariate data used for the analyses described below was collected at the initial evaluation; GPS driving indicators were based on 12 months of driving data generated by each subject, beginning with installation of the device at the time of the initial evaluation.

### Statistical analysis

Reported drug usage was summarized at the medication level (e.g., how often a drug in a given AHFS classification was reported in the brown bag review) and analyzed at the participant level (e.g., total medications used per participant, use or non-use of each medication class). Chi-square statistics were used to assess the relationship of use of each medication class with demographic factors and other covariates potentially associated with risk-related driving outcomes. Analysis of variance (ANOVA) was used to examine the bivariate relationships between medication use and the driving outcomes: speeding, rapid deceleration and right-to-left turn ratio.

Linear regression analysis was conducted to evaluate the relationship between medication use and the driving outcomes: speeding, rapid deceleration, and right-to-left turn ratio, while adjusting for covariates: age, gender, race/ethnicity, income, work status, marital status, and education level, urban/rural residence, study site, miles driven per year, and total driving days. Due to the skewed distributions of deceleration rate and speeding, these indicators were log-transformed in statistical analyses.

## Results

The LongROAD study recruited 2990 participants aged 65–79 years, with a mean age of 71 years. Approximately 20% of participants were recruited from each of the 5 study sites. At baseline, the sample was 47% male; 97% of participants had at least a high school diploma, including 23% with a bachelor’s degree and 40% with a master’s, professional or doctoral degree; 66% of participants were married, 15% were divorced and 13% widowed; 70% of the sample had a total household income of $50,000 or more, and 32% had an income of $100,000 or more; and 85% of participants were White Non-Hispanic; 71% lived in a metropolitan area and 30% reported working in the previous month at the time of evaluation. Brown bag data were available for 2949 participants (98.6%), and 2898 participants (96.9%) had both medication data and 30 or more recorded days of driving. Participants drove an average of 253 days during the first 12 months after GPS installation; of the mean annualized number of miles driven per year was 9437 miles per year (median 8408, standard deviation 5386); the mean of rapid deceleration events was 5.42 per 1000 miles driven (median 4.0, SD 6.38); the mean number of speeding events per 1000 miles driven was 7.83 (median 1.0; SD 17.27); the mean ratio of right-to-left turns was .93 (median 0.93, SD .13).

Of 24,697 medications recorded in the brown bag review, 22,863 (92.6%) were coded successfully in the AHFS classification system. Table 1 shows the frequencies by drug classifications for the Tier 1 AHFS categories, as well as the number and percentage of participants reporting at least one medication in each Tier 1 drug category. The most common categories were cardiovascular, vitamins, and CNS agents.

**Fig. 1:**
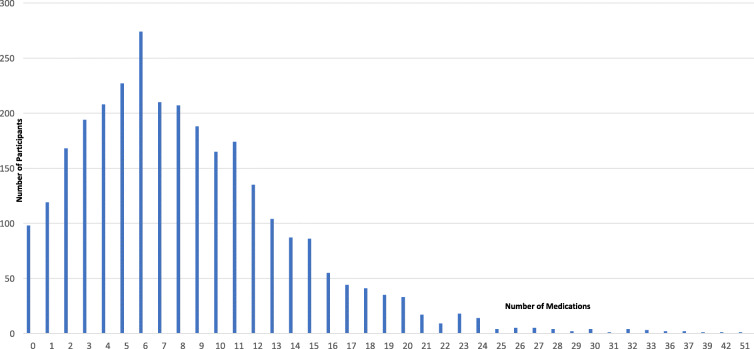
Frequency Distribution of Medications Used by Participants of the Longitudinal Research on Aging Drivers (LongROAD) Study (*n* = 2949)

Ninety-eight participants (3.3%) reported no medication use. The distribution of total medications taken (Fig. 1) was positively skewed: the median number of medications reported was 7; the maximum number of medications taken was 51.

Table 2 shows the distribution of the most frequently reported drugs, using the most detailed tier classification available for each medication. Electrolyte replacement preparations are the most commonly reported medication (*n* = 1624), followed by HMG CoA Reductase inhibitors (statins) (*n* = 1384), vitamin D (*n* = 1377), multivitamin preparations (*n* = 1298), and salicylates (*n* = 1293).

**Table 2:** Most common AFHS-classified medications reported: detailed tier specification^a^

Frequency of medications used (*N* = 24,697 medications)		
	N	%
40:12 Electrolyte Replacement Preparations	1624	6.6
24:06.08 HMG-CoA Reductase Inhibitors	1384	5.6
88:16 Vitamin D	1377	5.6
88:28 Multivitamin Preparations	1298	5.3
28:08.04.24 Salicylates	1293	5.2
88:08 Vitamin B Complex	789	3.2
24:24 beta-Adrenergic Blocking Agents	733	3.0
68:04 Adrenals	670	2.7
56:28.36 Proton-pump Inhibitors	647	2.6
24:32.04 Angiotensin-Converting Enzyme Inhibitors	608	2.5
24:06.92 Antilipemic Agents, Miscellaneous	590	2.4
68:36.04 Thyroid Agents	519	2.1
40:28.20 Thiazide Diuretics	516	2.1
28:08.04.92 Other Nonsteroidal Anti-inflammatory Agents	498	2.0
28:16.04.20 Selective Serotonin-reuptake Inhibitors	380	1.5
52:08 Anti-inflammatory Agents	354	1.4
28:08.92 Analgesics and Antipyretics, Miscellaneous	338	1.4
68:20.04 Biguanides	330	1.3
04:08 Second Generation Antihistamines	318	1.3
24:32.08 Angiotensin II Receptor Antagonists	317	1.3
52:08.08 Corticosteroids	304	1.2
24:28.08 Dihydropyridines	299	1.2
88:12 Vitamin C	299	1.2
12:12.08.12 Selective beta-2-Adrenergic Agonists	291	1.2
28:12.92 Anticonvulsants, Miscellaneous	241	1.0

^a^Excludes non-classified medications; the 25 most commonly reported medications are shown; denominator for percentages is total number of AHFS-classified medications (*N* = 22,863)

The distributions of medication use by age, sex and race/ethnicity are shown in Table 3. Of the 17 medication classes, usage increased significantly as a function of age in 8 classes, and there were significant gender differences in medication use for 12 of the 17 classes. However, there were only two medication class in which a significant race/ethnicity differences was observed: Black non-Hispanic participants had substantially higher rates of the electrolytic class of drugs, and Asian participants had significantly lower rates of CNS agents.

**Table 3:** Bivariate analysis for Gender, Age, and Race/Ethnicity

	Gender			Age				Race/Ethnicity					
Medication Class	Male (%) *N* = 1381	Female (%) *N* = 1564	p	65–69 (%) *N* = 1221	70–74 (%) *N* = 1024	75–79 (%) *N* = 700	p	White non-Hispanic (%) *N* = 2523	Black, Non-Hispanic (%) *N* = 207	Asian (%) *N* = 65	Other, Non-Hispanic (%)* N = 67	Hispanic (%) *N* = 79	P
Antihistamine	10.6	14.1	**<.01**	13.1	11.6	12.7	.56	12.7	11.1	9.2	17.9	8.9	.19
Anti-infective agents	9.3	10.4	.386	9.8	9.2	11.0	.46	10.0	9.7	4.6	11.9	8.9	.26
Anti-neoplastic agents	2.5	5.0	**<.01**	3.7	4.1	3.6	.82	3.9	3.4	1.5	4.5	2.5	.94
Autonomic	26.8%	17.3%	**<.01**	20.3%	22.6%	23.1%	.26	21.3%	25.1%	18.5%	28.4%	25.3%	.21
Blood Formation, Coagulation	17.9	9.1	**<.01**	9.6	14.1	18.4	**<.01**	13.3	14.5	13.8	9.0	12.7	.83
Cardiovascular	78.8	68.9	**<.01**	68.1	74.9	81.1	**<.01**	73.2	79.2	72.3	71.6	70.9	.58
CNS agents	71.0	69.6	.408	67.2	72.9	71.7	**<.01**	70.9	67.6	46.2	79.1	65.8	**<.01**
EENT	24.0	24.1	.968	21.8	24.6	27.3	**.02**	23.8	25.6	27.7	26.9	24.1	.84
Electrolytic	41.1	62.5	**<.01**	51.3	50.6	57.3	**.01**	52.5	61.8	44.6	44.8	41.8	**.02**
Gastrointestinal drugs	30.6	32.4	.32	29.5	32.3	34.0	.10	31.6	31.9	23.1	29.9	38.0	.45
Hormones and synthetic substitutes	38.2	50.1	**<.01**	43.6	44.4	46.3	.51	44.5	45.4	40.0	50.7	43.0	.62
Respiratory tract	3.0	4.1	.13	2.9	4.1	4.1	.12	3.4	4.8	7.7	3.0	5.1	.14
Skin and mucous membrane agents	18.0	15.0	**.03**	13.3	18.7	18.4	**<.01**	16.4	17.9	13.8	14.9	16.5	.92
Smooth Muscle Relaxants	1.9	4.0	<.**01**	2.0	3.3	4.4	**<.01**	3.0	2.9	1.5	3.0	6.3	.17
Vitamins	58.7	70.2	**<.01**	64.0	63.1	68.6	.05	65.6	60.9	50.8	65.7	63.3	.24
Not classified	32.1	38.7	**<.01**	33.4	36.7	37.7	.11	36.6	30.4	32.3	35.8	27.8	.22
Miscellaneous agents	15.7	10.2	**<.01**	11.1	13.1	15.3	**.03**	13.0	11.1	12.3	16.4	10.1	.79

Bivariate analyses (ANOVA) were conducted to determine the unadjusted relationship between each medication class and driving outcomes; findings are summarized in Table 4. Antihistamines were associated with higher right-to-left turn ratios. Eye, ear, nose and throat (EENT) agents, autonomic drugs, hormones, skin and mucous membrane agent, and local anesthetics were associated with more rapid decelerations. Only one class, electrolyte, caloric and water balance medication, was associated with a *lower* incidence of rapid deceleration and speeding.

**Table 4:** Unadjusted Risk-Related Driving Indicators by Tier 1 Drug Class: Mean (Standard Deviation)

Drug Class (N Taking Drug)	Deceleration Rate^**a**^		p	Speeding Rate^**b**^		p	Right/Left turn Ratio^**c**^		p
Use of Drug:	No	Yes		No	Yes		No	Yes	
Antihistamine (396)	5.4 (6.2)	5.7 (7.2)	.33	7.6 (16.4)	9.27 (21.8)	.07	.93(.13)	.95(.14)	**.01**
Anti-infective (295)	5.4 (6.4)	5.5 (5.9)	.76	7.8 (17.6)	8.2 (14.5)	.73	.93(.13)	.94(.13)	.89
Anti-neoplastic (111)	5.4 (6.3)	7.7 (17.1)	.10	7.7 (17.1)	10.7 (21.1)	.07	.94(.13)	.95(.14)	.42
Autonomic drugs (648)	5.3 (6.0)	6.0 (7.6)	**.01**	7.6 (16.5)	8.8 (19.7)	.12	.93(.13)	.94(.13)	.41
Blood formulation, coag, thrombosis (389)	5.3 (6.3)	6.0 (7.0)	.08	7.9 (17.7)	7.2 (13.9)	.43	.94(.13)	.94(.15)	.90
Cardiovascular drugs (2146)	5.5 (6.1)	5.4 (6.5)	.69	8.3 (18.6)	7.7 (16.8)	.43	.94(.13)	.94(.13)	.80
CNS agents (2051)	5.2 (6.3)	5.5 (6.4)	.23	7.1 (16.0)	8.1 (17.8)	.13	.93(.14)	.94(.13)	.23
Electrolytic, Caloric and Water Balance (1601)	5.8 (7.2)	5.1 (5.6)	**<.01**	8.7 (18.7)	7.1 (16.0)	**.01**	.94(.13)	.94(.13)	.86
Respiratory Tract Agents (105)	5.4 (6.4)	6.0 (6.2)	.34	7.6 (17.2)	9.8 (20.0)	.23	.94(.13)	.93(.13)	.81
Eye Ear Nose and Throat Preparations (718)	5.2 (6.1)	6.0 (7.1)	**<.01**	7.9 (17.0)	7.7 (18.0)	.82	.93(.13)	.94(.13)	.13
Gastrointestinal drugs (944)	5.3 (6.4)	5.6 (6.4)	.21	7.6 (16.4)	8.4 (19.0)	.25	.93(.13)	.94(.13)	.20
Hormones and Synthetic Substitutes (1300)	5.1 (6.0)	5.9 (6.8)	**<.01**	7.4 (16.0)	8.4 (18.7)	.10	.93(.13)	.94(.13)	.17
Local anesthetics (23)	5.4 (6.2)	10.0 (17.6)	**<.01**	7.8 (17.2)	11.6 (24.2)	.30	.94(.13)	.96(.14)	.47
Skin and Mucous Membranes (484)	5.3 (6.1)	6.0 (7.6)	**.03**	7.7 (16.9)	8.6 (18.8)	.30	.93(.13)	.94(.13)	.45
Smooth Muscle Relaxants (89)	5.4 (6.4)	5.5 (5.1)	.86	7.9 (17.4)	6.8 (13.9)	.55	.94(.13)	.94(.13)	.58
Vitamins (1899)	5.6 (6.7)	5.4 (6.2)	.41	8.3 (18.6)	7.6 (16.5)	.27	.94(.13)	.94(.13)	.91
Miscellaneous agents (371)	5.4 (6.5)	5.5 (5.6)	.85	7.6 (16.7)	9.5 (20.5)	.05	.93(.13)	.95(.13)	.10

^a^ Number of high deceleration (≥0.35 g) events events per 1000 miles driven

^b^ Number of speeding (> 80 mph) events per 1000 miles driven

^c^ Ratio of right turns to left turns; a higher mean ratio is indicative of possible reluctance to make left turns against oncoming traffic

In order to assess the effect of each medication on driving outcomes adjusting for potential confounders and covariates, a preliminary set of regression analyses was run to determine the relationship between each covariate on each outcome, adjusting for the effect of the other covariates. The results (not shown) indicated that total medications, miles driven per year, male gender, being non-white, living outside a metropolitan area and high income were independently associated with increased rapid deceleration events (*p* < .02). Older drivers had an increased right-to-left turn ratio (*p* < .001), indicating more cautious driving behavior; those with high incomes and a lower right-to-left turn ratio (*p* = .007). There were significant differences between study sites with respect to all three of the driving outcomes.

The results of linear regression analyses assessing the effect of each medication on the three driving outcomes, adjusting for all of the covariates described above, are shown in Table 5. As in the bivariate analyses, antihistamine use was significantly associated with an increased right-to-left turn ratio, the indicator of left turn avoidance (*p* = .016). CNS agents were significantly related to greater speeding incidence (*p* = .004). Use of hormones and gastrointestinal agents were significantly associated with increased rapid deceleration (*p* = .024 and *p* = .032); an increase in total medications used was also associated with increased rapid deceleration (*p* = .001). And as in the bivariate analyses, electrolytic agents were related to reduced rapid deceleration, after adjustment for all covariates.

**Table 5:** Model-based Relationship (B) between Medication Class and Driving Outcomes, Adjusted for Covariates^a^

	Speeding		Rapid Deceleration		Right-to-Left Turn Ratio	
Medication Class	B	p-value	B	p-value	B	p-value
Antihistamine	−.018	.790	−.010	.779	**.016**	**.016**
Anti-infective agents	.059	.424	−.027	.515	−.012	.096
Anti-neoplastic agents	.221	.054	.058	.376	.008	.469
Autonomic	.024	.670	.052	.099	.005	.394
Blood Formation, Coagulation	−.079	.242	.021	.586	−.003	.674
Cardiovascular	−.031	.561	−.055	.068	−.004	.487
CNS agents	**.143**	**.004**	.040	.165	.040	.165
Electrolytic	−.078	.105	**−.110**	**<.001**	.000	.933
EENT	−.075	.164	.046	.139	.003	.583
Gastrointestinal drugs	.058	.242	**.061**	**.032**	.006	.219
Hormones and synthetic substitutes	.054	.252	**.060**	**.024**	.003	.562
Local anesthetics	−.002	.993	.133	.350	.001	.966
Skin and mucous membrane agents	−.005	.936	.029	.423	−.005	.454
Smooth Muscle Relaxants	.007	.957	−.020	.779	−.002	.893
Vitamins	−.021	.675	−.034	.228	−.010	.053

^a^Covariates: total number of medications, miles driven per year, total days driving, sex, age, race/ethnicity, education, urban/rural residence, income, employment, study site

## Discussion

This study identified relationships between medication use and driving safety-related behaviors. CNS agents, hormones, and gastrointestinal medications were the medication categories significantly associated with risky driving behaviors, as was the total number of medications used, while electrolyte use was associated with lower high-risk driving behavior. The mechanisms underlying these relationships is unclear. The association between CNS agents, which includes analgesics, and speeding suggests possible mechanisms such as agitation, poor judgment and concentration. Electrolyte formulations may reduce rapid deceleration (hard braking) via improved muscle contractility and blood pressure regulation. Higher right-to-left turn ratios with antihistamines may reflect driving insecurity due to side effects, which may include drowsiness, lethargy, blurred vision, and impaired concentration. Further research is needed to better understand the mechanisms.

It should be emphasized that the underlying relationships between driving, the diseases being treated, and the medications themselves are complex and it is difficult to distinguish the effect of a medication from effects caused by the underlying medical condition that the medication is being used to treat. Regardless, use of certain medications, as well as total number of medications, can be considered indicators of increased driving risk and may help researchers and clinicians identify higher-risk populations who may benefit from intervention. The high usage of medications found in in this study’s ‘brown bag’ review of older drivers is consistent with rates reported in previous studies. For example, Qato et al. found that among community dwelling older adults in New Zealand, 81% used at least one prescription medication, and 29% used at least 5 prescription medications (Qato et al. 2008). Narayan et al. found that for older adults with an average age of 74.7 years who were receiving at least one prescription medication, the mean number of dispensed medications was 5.4 (Narayan and Nishtala 2015).

Medications taken by a high percentage of the study population included cardiovascular drugs (73%), vitamins (65%), CNS agents (70%), electrolytes (52%) and hormones (44%). In comparison, Gurwitz et al., in a 2003 cohort study over a 12 month period of 28,000 Medicare+Choice employees, found cardiovascular drugs in 53%, antibiotics in 44.5%, diuretics in 29.5%, lipid-lowering drugs in 22%, and opioid analgesics in 19% (Gurwitz et al. 2003). The higher rates of antibiotics in the Gurwitz et al. studies likely reflect the 12-month data collection period, as opposed to the single ‘point in time’ that medication data were collected for our study (at the baseline intake visit).

The high number of drugs taken by this cohort of older drivers has implications for physicians and pharmacists treating older drivers, with 18.5% of the medications included in the 2015 Beers Criteria of potentially inappropriate drugs (By the 2019 American Geriatrics Society beers criteria® update expert panel 2019; Li et al. 2019) . Studies of older drivers and physician counseling about medications and driving have demonstrated a lack of knowledge on the part of both patient and physician. A 2009 AAA Foundation for Traffic Safety study found that only 17.6% of drivers 55 years and older had received a warning from a health-care provider about the possible effect of medications on their ability to drive; only a slightly higher percentage of those on five or more medications received a warning about driving (18.8%) (MacLennan et al. 2009). And in a study of older drivers presenting to the emergency room who had driven in the last 30 days, none of those using sedating medications reported receiving counseling about driving (Henderson et al. 2016).

We believe that the AHFS classifications, which are now an integral part of the study database, will have great value in illuminating the factors affecting the driving behavior of older adults, as well as the risks that this population experience with advancing age. Statistics summarizing the use of medications in terms of major Tier 1 AHFS categories have been provided in this paper; more detailed Tier 2, 3 and 4 categorizations as well as doses for each medication have also been coded and stored in the main project database for use in ongoing research. This database will support the study of subclasses of drugs within the tiers, such as the CNS drugs, which include analgesics, anxiolytics, and anti-parkinsonian drugs. Detailed analysis of medication will also allow application of drug burden indices, which can be used to measure the impact of medications on functional status (Hilmer et al. 2007). Additionally, our access to medical records will allow an analysis of the effects of medications with respect to disease control.

The findings of the LongROAD study, as well as the medication usage data presented here, may inform the work of policy-makers, who may commission and support similar methodology for future studies involving older drivers and medication usage. Population-based and system-level interventions, including legislation, will be needed to address poly-pharmacy in general, and issues relevant to older adults specifically. Policy makers and practitioners alike may apply these findings to identify high-risk drivers – review of medication lists may prompt practitioners to re-evaluate the appropriateness of each medication, the patient’s underlying medical conditions, and overall fitness to drive. While crashes in older adults are less than younger drivers, the elderly are at higher risk of severe injury and death, and interventions to reduce the risk of crash to as low as possible is especially important in this group.

This study included a number of limitations. While LongROAD’s multi-site study sample has substantial representation in terms of gender and age among those 65 years and older, the study participants are predominantly white and well-educated. Given recruitment in health care settings, participants may also be greater users of health care (though data show that nearly 95% of US adults 65 years of age or older report having a personal doctor or health care provider) (Centers for Disease Control and Prevention - Division of Population Health 2017). This cohort is also relatively healthy, as inclusion criteria include non-institutional dwelling, active driving status, and no significant cognitive impairment at baseline. Additionally, driving-related analysis is based on baseline medication review, and participants may have changed their medications during the course of the study. Strengths of this study include the objective assessment of medications and driving behavior for a large number of participants, whereas many studies this size rely on self-reporting for either or both of these measures, and the recruitment of participants from diverse geographic locales across the US, including both rural and urban communities. Prior studies have often been limited to crash-outcomes analysis. The GPS analysis used in this study permits the identification of the more common potentially risky driving behaviors. While this study was limited to drivers 65 years and older, the information on medication effects on driving will guide further research on driving across all ages. Subsequent analyses of LongROAD data, as we continue to evaluate the cohort in annual assessments, will provide further insight into the effect of medications on driving behavior and safety.

## Conclusion

Several classes of medications were associated with objective indicators of unsafe driving.

## Data Availability

The datasets used and/or analyzed during the current study are available from the corresponding author on reasonable request.

## Abbreviations

AHFS: American Hospital Formulary Service
ANOVA: Analysis of variance
CNS: Central Nervous System
MVC: Motor Vehicle Crash
OTC: Over the counter
